# Circulating immune complexome analysis identified anti-tubulin-α-1c as an inflammation associated autoantibody with promising diagnostic value for Behcet’s Disease

**DOI:** 10.1371/journal.pone.0199047

**Published:** 2018-06-14

**Authors:** Yongjing Cheng, Xiaozhen Zhao, Yuling Chen, Yuhui Li, Rulin Jia, Lei Zhu, Cibo Huang, Xiaolin Sun, Haiteng Deng, Zhanguo Li

**Affiliations:** 1 Department of Rheumatology and Immunology, Peking University People's Hospital and Beijing Key Laboratory for Rheumatism Mechanism and Immune Diagnosis Beijing, China; 2 Department of Rheumatology and Immunology, National Center of Gerontology, Beijing Hospital, China; 3 MOE Key Laboratory of Bioinformatics, School of Life Sciences, Tsinghua University, Beijing, China; Duke University School of Medicine, UNITED STATES

## Abstract

**Background:**

Behcet’s disease (BD) is a chronic, multisystem-involved vasculitis and its pathogenesis remains elusive. No specific serological markers for BD diagnosis have been established. Identification of novel diagnostic biomarkers will be helpful in timely diagnostic and treatment for Behcet’s disease.

**Objective:**

To screen novel autoantigens or autoantibodies with potential diagnostic value in circulating immune complexes (CICs) from BD patients.

**Methods:**

A proteomic strategy for immune complexome analysis was developed, in which CICs were separated from serum sample of 10 BD patients and 10 healthy controls and then subjected to Orbitrap mass spectrometry for autoantigen profiling. Anti-tubulin-**α**-1c antibody levels were further determined by enzyme-linked immunosorbent assay (ELISA) in sera of patients with BD, systemic lupus erythematosus (SLE), recurrent aphthous ulcers (RAU), ANCA associated systemic vasculitis (AASV), Takayasu's arteritis (TA) and 59 healthy controls.

**Result:**

A total of 17 potential antigens were identified in CICs from BD patients, but not in HC. The autoantibody to one of the identified antigens, tubulin-**α**-1c, was significantly increased in BD patients compared with that in healthy and disease controls. The sensitivity and specificity of tubulin-**α**-1c antibody in the diagnosis of BD in this study were 61.36% and 88.4%, respectively. Further analysis demonstrated that anti-tubulin-**α**-1c was associated with complications of deep venous thrombosis and erythema nodosum in BD. The levels of anti-tubulin-α-1c were also significantly correlated with the BD inflammation and disease activity markers ESR, CRP and BVAS.

**Conclusion:**

Anti-tubulin-**α**-1c antibody is a promising biomarker in diagnosis and severity evaluation of BD and in indicating the risk of deep venous thrombosis and erythema nodosum. The immune complexome analysis by proteomic CIC autoantigen screening is a feasible way of identifying novel biomarkers in BD.

## Introduction

Behcet’s Disease (BD) is an autoimmune vasculitis with multi-organ involvement, characterized by recurrent occurrence of oral and genital ulcer, skin lesions, and ophthalmological, neurological, or gastrointestinal manifestations. The disease is particularly prevalent in regions along the “Silk Road”, extending from East Asia to the Middle Eastern and the Mediterranean countries. The highest prevalence of the disease is only 14–20/100 000 along the Silk Road, and is much more rarely seen in Western countries [1–3]. Since efficient characteristic laboratory tests are absent, the diagnosis of BD is clinical and upon an expert opinion [4]. Early diagnosis, close monitoring, early and appropriate treatment is mandatory to reduce morbidity and mortality of this disease [4]. However, the lack of reliable serological markers for accurate diagnosis of BD restrains the improvement of BD treatment in clinical practice [5–7]. Therefore, identification of novel biomarkers for BD diagnosis is in need for better management of BD.

Recently, a variety of autoantigens have been reported in BD, including vascular proteins such as α-enolase[8], organor tissue original proteins such as alpha-tropomyosin and selenium binding protein[9, 10, 11] and environmental pathogenic factors which may cause cross-reactivity to human antigens[12]. However, due to limited sensitivity and specificity of recently identified autoantigens as biomarkers, many BD patients are still suffering from delayed diagnosis and treatment.

Circulating immune complexes (CICs) [13] present in peripheral blood contain a variety of antigens that may associate with underlying diseases. Autoantigens incorporated into CICs are promising candidates for diagnostic biomarker screening. In this study, we applied high resolution mass spectrometry with nano-LC combined with Orbitrap Q Exactive mass spectrometer to identify potential BD autoantigens in CICs present in BD patients. A total of 17 novel potential autoantigens were identified from CICs of Behcet’s Disease. The autoantibody against one of these autoantigens, tubulin-α-1c, was further studied and might be a novel diagnostic biomarker with promising sensitivity and specificity in BD. The anti-tubulin-**α**-1c antibodies were associated with BD inflammation level and disease activity and may participate in the pathogenesis of the disease.

## Materials and methods

### Patients

The study was approved by the Ethics Committee of Peking University People’s Hospital (Approval No. 2014PHB087-03). All participants recruited in this study provided informed written consent for participation in this study. Authors had access to information that could not identify individual participants during or after data collection. Serum samples were obtained from 101 patients admitted to Department of Rheumatology and Immunology, Peking University People’s Hospital, between November 2016 and October 2017. 44 patients with BD (mean age 40.49±15 years, 28 males and 16 females), 32 patients with RAU (mean age 32.68±9.42 years, 7 males, 25 females), 25 patients with AASV (age 63.77±7.99 years, 17 males, 8 females), 51 patients with SLE (age 38.69±13.77 years, 8 males, 43 females), and 13 patients with TA (age 33.38±10.15 years, 1 males, 12 females)were enrolled in the Enzyme-Linked Immunosorbent Assay (ELISA) validation study. Serum samples from age- and sex-matched healthy donors were used as a negative control. Health controls, who were age and sex matched volunteers and were excluded the possibility to have autoimmune disorders, also signed informed consents. BD was diagnosed by the international criteria in 1990 [14], and AASV were diagnosed according to the Chapel Hill Nomenclature Conference patients criteria in 1994[15]. There was no significant difference in gender and age between BD patients and healthy controls. All serum samples were obtained with informed consents and the study was approved by the ethics committee of Peking University People’s Hospital. For BD patients, we calculated the Birmingham Vasculitis Activity Score (BVAS) [16,17] to evaluate the overall disease activity of BD.

### Proteomic analysis and ELISA experiment

#### 1. Immunoprecipitation

Pooling serum samples were collected from10BDpatientsand10 healthy controls and pooled together respectively. CICs were purified by protein G PLUS–Agarose beads (Santa Cruz: SC-2002). Briefly, Beads (50μl) were incubated with 30μl of pooled serum sample on a rotator at room temperature for 120 minutes. The beads with bound ICs were collected by centrifugation (1000 G, 10min)at 4°C, and the supernatants were aspirated. The beads were washed 3 times with 1 ml PBS. After the final wash, the supernatants were discarded and the beads were suspended in 40 μl of electrophoresis sample buffer. Then the samples were boiled for 3 minutes and subjected to SDS-PAGE and subsequent gel staining.

#### 2. In-gel trypsin digestion

Each lane was cut into 10 pieces, then the 25kd and 75kd piece of lane were removed, the rest were reduced with 25 mM of Dithiothreitol (DTT) and alkylated with 55 mM Iodoacetamide. Then in gel digestion was carried out with sequencing grade modified trypsin (Promega, Fitchburg, WI) in 50 mM ammonium bicarbonate at 37°C overnight. The supernatants containing the peptide digests of CIC components were extracted twice with 1% trifluoroacetic acid in 50% acetonitrile aqueous solution for 30 minutes. The extractions were then centrifuged in a speed vac to reduce the volume to approximately 80 ul.

#### 3. LC-MS/MS analysis

The peptide mixture (10ul) was subjected to LC-MS/MS with nano-LC combined with Orbitrap Q Exactive mass spectrometer. The raw data were searched again stipi. HUMAN.v3.84 database using Protein Discovere1.3.0. The searched parameters were set below: peptide ms tolerance as 20 ppm; ms/ms tolerance as 20 mmu.

#### 4. Expression of recombinant human tubulin-α-1c protein

The cDNA encoding human tubulin-**α**-1c was amplified by RT-PCR from total RNA of human whole peripheral blood samples. The nucleotide sequences of the primers were as follows: sense 5'-CGCGGATCCATGCGTGAGTGCATCTC-3' and antisense 5'-CCGCTCGAGATACTCTTCACCCTCATCCTCTCC-3'. The amplified tubulin-**α**-1c cDNA was subcloned into the expression vectorPET30a. The 58kDa recombinant human tubulin-**α**-1c was expressed in Escherichia coli DH5a and was purified by the histidine-Ni affinity purification system and was soluted in buffer containing 8 M urea and 100 mM imidazole.

#### 5. Enzyme-linked immunosorbent assay (ELISA)

Serum concentrations of anti-tubulin-**α**-1c antibody were determined by ELISA. Briefly, 96 well polysorp plates (NUNC, Denmark) were coated with recombinant tubulin-**α**-1c protein at a concentration of 10μg/ml in 0.05 M carbonate buffer at 4°C overnight. The wells were then washed with PBS with 0.05% Tween-20 (PBS-T) four times and blocked with 3%BSA-PBS for 3.5 hours at 37°C. Serum samples were diluted at 1:100 with PBS-T containing 1% BSA and were added to 96-well plate. Wells filled only by PBS-T containing 1% BSA without human serum samples were set up to examine non-specific background. After incubation 1.5h at 37°C, all the wells were washed for six times with PBS-T. Then, 100ul of goat anti-human IgG conjugated to peroxidase, diluted at 1:5000 in PBS-T, was added to each well and incubated for 30 minutes at 37°C. After washing with PBS-T four times, the bound antibodies were detected with o-phenylenediamine (OPD) as substrate. The reaction was stopped by adding 100ulof 2Msulfuric acid to each well. Absorbance density (OD) was determined at 450nm by a Bio-Rad plate reader. The values of OD of anti-tubulin-α-1c were transformed to arbitary units (AU), calculated as below:

$$AU=\frac{[OD\;(peptide)-OD\;(nonspecific\;background)]test\;serum}{[OD\;(peptide)-OD\;(nonspecific\;background)]positive\;control\;serum}\times 100$$

An AU value greater thanmean+2×S.D. of the normal control sera was considered positive.

#### 6. Clinical and laboratory data of BD patients

The following clinical and laboratory data were collected from the medical record database of Peking University People’s Hospital: Oral ulcer, cancrum pudenda, fever, Ophthalmitis, Erythema nodosum, Intestinal BD,DVT, Arthritis, anti-AECA antibody, rheumatoid factor (RF), immunoglobulins (IgG, IgM, IgA), erythrocyte sedimentation rate (ESR), and C-reactive protein (CRP). Anti-AECA antibody was detected with an indirect immunofluorescence (IIF) kit (Euroimmun, Germany). ESR was measured by the Westergren method, and≤15 mm/h for males and ≤20 mm/h for females were considered as normal. CRP and immunoglobulins were examined by immunonephelometry. Values>7.9 mg/L for CRP was considered positive. Normal ranges of IgG, IgM, and IgA were 6.94–16.1, 0.60–2.63, and 0.68–3.78 g/L, respectively. The Birmingham Vasculitis Activity Score (BVAS) was calculated as previously described [16, 17]

#### 7. Data analysis

Data analyses were performed using SPSS 17.0 for Windows. Results are presented as the mean ± SD and percentage. Quantitative data were compared by Mann-Whitney test. Spearman correlation analysis were used for correlation analysis. A difference between groups was considered significant if p < 0.05.

## Results

### 2.1 Identification of autoantigens incorporated into CICs in Behcet’s Disease by high resolution mass spectrometry

The proteins identified by immune complexome analysis of BD patients and healthy controls are summarized in Table 1. An antigen profile composed of 423 proteins was identified from CICs of both BD patients and healthy controls. 17 proteins were specifically identified in CICs from BD patients, but not in CICs from HC (Table 1). These potential autoantigens could be divided into different groups below, based on their function: immune response (V3-4 protein, anti-streptococcal/anti-myosin immunoglobulin lambda light chain variable region, lysozyme C); cell mobility, structure, or cell integrity (Cingulin, BAG family molecular chaperone regulator 3 (BAG3), tubulin alpha-1C); lipid transportation (isoform 2 of cholesteryl ester transfer protein, apolipoprotein L1 isoform c precursor),regulation of transcription (zinc finger protein 669, α-enolase); extracellular matrix integrity (Isoform E of Proteoglycan 4), intracellular signal transduction (Serine/threonine-protein kinase TAO3 (TAOK3), retinal guanylyl cyclase 1) and the coagulation cascade (Vitamin K-dependent protein S, protein Z-dependent protease inhibitor, Kininogen-1). The remaining proteins were ankyrin repeat domain-containing protein 34A, whose functions were still elusive. Among these proteins, α-enolase has been previously proved to be an autoantigen of BD [8], and autoantibodies againstα-enolase were identified as components of anti-endothelial cell antibody (AECA). Since AECA was a diagnostic biomarker commonly applied for BD and other vasculitis [8], the identification ofα-enolase supported the feasibility of proteomic screening on CICs for BD diagnostic biomarkers in our study.

**Table 1:** Potential autoantigens identified in CICs inform patients with BD by high resolution mass spectrometry.

IPI number	Protein name	Ms sore
IPI00465248.5	Isoform alpha-enolase of Alpha-enolase	3.56
IPI00914948	apolipoprotein L1 isoform c precursor	14
IPI00027242	Retinal guanylyl cyclase 1	14
IPI00007199	Protein Z-dependent protease inhibitor	17
IPI00019038	Lysozyme C	18
IPI00917199	zinc finger protein 669 isoform 2	19
IPI00878131	Vitamin K-dependent protein S	21
IPI00852984	Cingulin	22
IPI00410485	Serine/threonine-protein kinase TAO3 (TAOK3)	23
IPI00387144	Tubulin alpha-1C	23
IPI00465409	Ankyrin repeat domain-containing protein 34A	24
IPI00641582	BAG family molecular chaperone regulator 3 (BAG3)	24
IPI00656111	Isoform E of Proteoglycan 4	27
IPI00641481	Isoform 2 of Cholesteryl ester transfer protein	37
IPI00477714	V3-4 protein	44
IPI01012492	Kininogen-1	48
IPI00956602.1	Anti-streptococcal/anti-myosin immunoglobulin lambda light chain variable region	112

### 2.2 Anti-tubulin-α-1c autoantibody was significantly increased in BD patients

**Fig 1:**
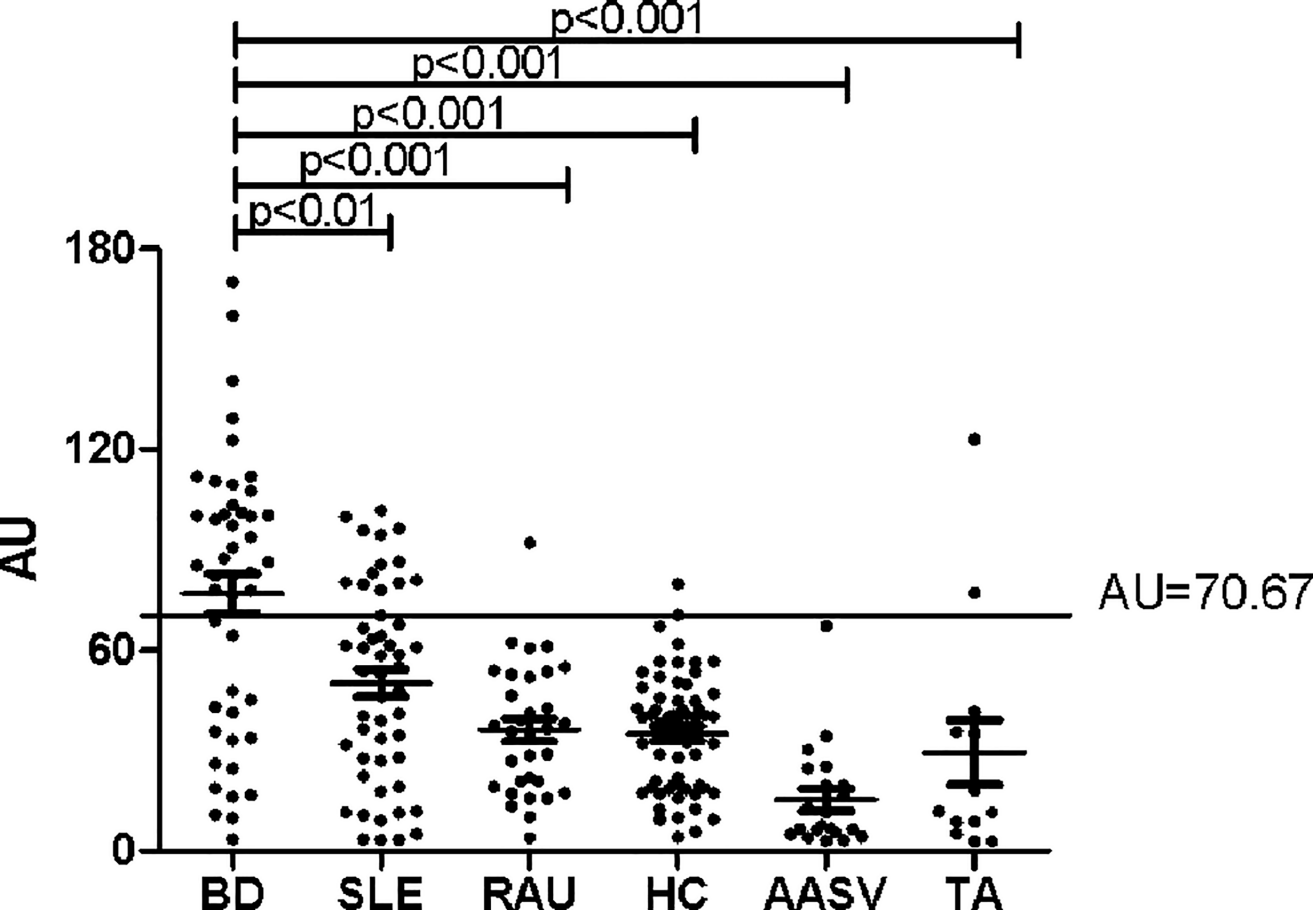
Anti-tubulin-α-1cantibody levels in patients and healthy controls. The sensitivity and specificity of tubulin-α-1c antibody in the diagnosis of BD were 61.36% and 88.4% respectively. (BD 44, SLE 51, RAU 32, HC 59, AASV 25, TA 13).

To further validate the reliability and diagnostic significance of the potential autoantigens screened in CICs, we selected tubulin-**α**-1c as a candidate autoantigen for further validation. Different members of tubulin family have been identified as autoantigens in several diseases with autoimmunity such as GVHD [18–22], which indicated that tubulin molecule could become autoantigens in autoimmune conditions. Whether there are autoreactivity with tubulin members in BD is still elusive. By indirect ELISA, we evaluated the levels of autoantibodies against tubulin-α-1c in serum from patients with BD, healthy controls and patient controls with recurrent aphthous ulcers (RAU), ANCA associated systemic vasculitis (AASV),Takayasu's arteritis(TA)and systemic lupus erythematosus (SLE), who needed to be distinguished from BD in clinical practice. As shown in Fig 1, levels of anti-tubulin-α-1c antibodies were significantly elevated in BD patients in comparison with healthy controls and disease controls (AU: BD vs. SLE: 72.80±37.5 vs. 50.27±29.45, p<0.01; BD vs. RAU: 72.80±37.5 vs. 36.19±19.41, p<0.001; BD vs. HC: 72.80±37.5 vs. 35.13±17.67, p<0.001: BD vs. AASV: 72.80±37.5 vs. 15.34±15.22, p<0.001: BD vs. TA: 72.80±37.5 vs. 29.48±35.19, p<0.001).

### 2.3 Anti-tubulin-α-1c autoantibody was a promising biomarker for BD diagnosis

A cut-off value (AU: 70.67) was determined by the mean value plus two times standard deviation of anti-tubulin-α-1c levels in healthy controls to group BD patients into one group with abnormally elevated anti-tubulin-α-1c levels and another with normal anti-tubulin-α-1c levels, With this cut-off value, the diagnostic power of anti-tubulin-α-1c in BD was evaluated, the positive rate of anti-tubulin-α-1c antibody in BD was 56.8%, significantly higher than SLE(27.5%, p<0.01), RAU(3.11%, p<0.01), AASV (4%,p<0.01), TA (23.1%,p<0.01)and HC(3.39%,p<0.01). More than half of the BD patients recruited in this study were positive for anti-tubulin-α-1c, while much rarer positive cases were found in the control groups and the sensitivity and specificity of anti-tubulin-α-1c in BD diagnosis were 61.36% and 88.4% respectively (Table 2). This result showed that anti-tubulin-α-1c might be a promising biomarker for BD diagnosis.

**Table 2:** Prevalence of anti-tubulin-α-1c in BD patients and controls.

group	number	AU	anti-tubulin-α-1C antibody		
			positive cases	Sensitivity(%)	Specificity(%)
BD	44	72.80±37.5	27	61.36	88.4
SLE	51	50.54±29.7	14	27.5	-
RAU	32	36.14±19.1	1	3.11	-
HC	59	35.13±17.67	2	3.39	-
AASV	25	15.34±15.22	1	4	-
TA	13	29.48±35.19	3	23.1	-

BD, Behcet’s Disease; AASV, ANCA associated systemic vasculitis; RAU, recurrent aphthous ulcers; HC, healthy control; TA: Takayasu's arteritis; SLE: systemic lupus erythematosus.

Anti-endothelial cell antibody (AECA) was the diagnostic biomarker for BD widely applied in current clinical practice. The diagnostic efficiency of AECA in BD was controversial in different studies, and the reported sensitivity of AECA in BD diagnosis ranged from 26% to 48% and the specificity was from 43% to 68% [23–26]. Several novel autoantigens recently identified such asα-enolase were proved to be components of the AECA autoantigens [8]. To elucidate whether anti-tubulin-α-1c was also components of AECA, we compared the overlapping of BD patient groups positive for anti-tubulin-α-1c and AECA. There was 29% of BD patients positive for both anti-tubulin-α-1c and AECA, much less than that of patients positive for only anti-tubulin-α-1c (61.36%) or AECA (47.2%), which implicated that anti-tubulin-α-1c might not be a part of AECA.

We further compared the diagnostic performance of anti-tubulin-α-1c and AECA (Table 3). The sensitivity and specificity of anti-tubulin-α-1c in BD diagnosis were 61.36% and 88.4% respectively, which were both superior to the performance of AECA (sensitivity 47.2% and specificity 66.1%). Since anti-tubulin-α-1c and AECA were not totally overlapped, we further evaluate the diagnostic power of the combinative application of anti-tubulin-α-1c and AECA. The results showed that the single test of anti-tubulin-α-1c in BD diagnosis was superior to the combination of anti-tubulin-α-1c and AECA as well as AECA alone (Table 3), which suggested that anti-tubulin-α-1c could be a promising biomarker for BD diagnosis.

**Table 3:** The performance of anti-tubulin-α-1c antibodies and anti-endothelial cell antibodies in BD diagnosis.

	Sensitivity(%)	Specificity(%)
anti-tubulin-α-1c antibodies	61.36	88.40
AECA	47.17	66.11
anti-tubulin-α-1c antibodies and AECA	28	92.4
anti-tubulin-α-1c antibodies or AECA	70	69.4

AECA, anti-vascular endothelial cell antibodies.

### 2.4 Anti-tubulin-α-1c autoantibody was associated with BD disease activity and vascular damage

**Fig 2:**
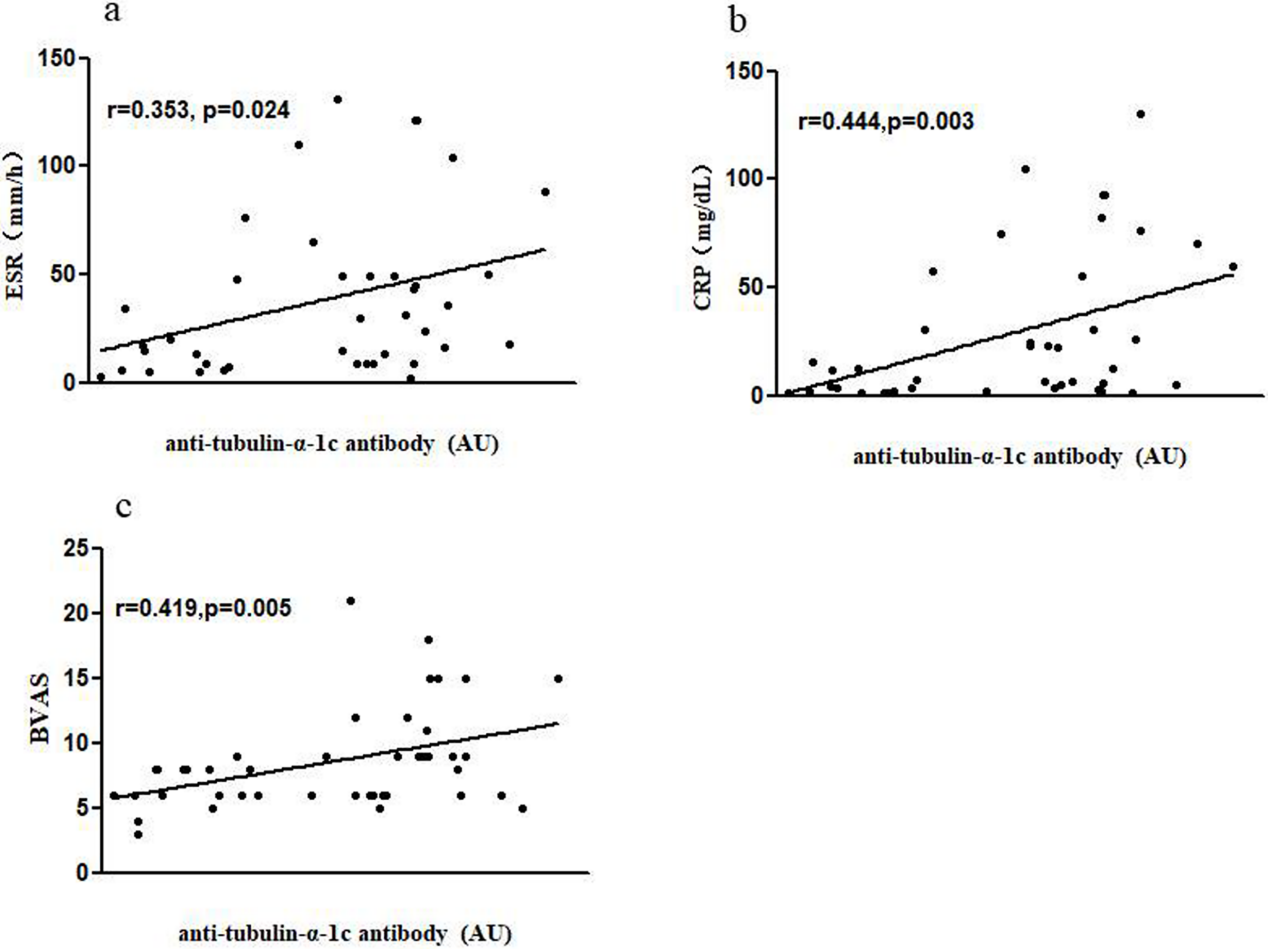
Correlation of serum anti-tubulin-α-1c antibody level and disease activity index in BD patients. (a) anti-tubulin-α-1c antibody level positively correlated with ESR; (b) anti-tubulin-α-1c antibody level positively correlated with CRP; (c) anti-tubulin-α-1c antibody level positively correlated with BVAS. BVAS, Birmingham vasculitis activity score.

Anti-tubulin-α-1c autoantibody was also a biomarker for disease activity of BD. Correlation analysis showed that anti-tubulin-α-1c levels were positively correlated with BD disease activity markers ESR(r = 0.354, p<0.05) and CRP (r = 0.444, p<0.05) (Fig 2 and Table 4). To directly evaluate the indicative value of anti-tubulin-α-1c on the overall vasculitis activity of BD, we calculated the Birmingham Vasculitis Activity Score (BVAS)[16,17]and analyzed its correlationship with anti-tubulin-α-1c. As shown in Fig 2, anti-tubulin-α-1c was significantly correlated with BVAS(r = 0.419, p<0.05).No correlation were found between levels of anti-tubulin-α-1c and serum levels of IgG, IgA, and IgM. The ages of BD patients were not correlated with anti-tubulin-α-1c levels, either (r = 0.079, p = 0.641).

**Table 4:** Correlation of serum anti-tubulin-α-1cantibodies with clinical features of RA patients.

Parameter	Spearman correlation coefficient	P value
ESR	**0.353**	**0.024**
CRP	**0.444**	**0.003**
IgG	0.314	0.058
IgA	0.054	0.752
IgM	0.160	0.350
RF	0.114	0.503
C3	0.075	0.663
C4	0.242	0.156
BVAS	**0.419**	**0.005**

To further elucidate the clinical relevance of anti-tubulin-α-1c antibody in BD, we divided BD patients into anti-tubulin-α-1c positive and anti-tubulin-α-1c negative groups. The incidence of BD clinical manifestations including oral ulceration, fever, ophthalmitis, intestinal BD, erythema nodosa, vasculitis related autoantibody AECA and autoimmunity associated autoantibody ANA were compared between these two groups (Table 5). The presence of anti-tubulin-α-1c was significantly associated with complications of deep venous thrombosis and erythema nodosum in BD patients (Table 5), which suggested anti-tubulin-α-1c might take part in the vascular damage process of BD. We also compared the anti-tubulin-α-1c levels between male and female BD patients and no difference were observed between the male and female (28 males(anti-tubulin-α-1cAU: 58.99±27.92), 16 females(anti-tubulin-α-1c AU: 53.05±28.01),p = 0.532).

**Table 5:** Phenotype difference of BD patients who are anti-tubulin-α-1c positive and negative.

	Anti-tubulin-α-1cAntibody(+) (n = 26)n (%)	Anti-tubulin-α-1c Antibody(-) (n = 18)n (%)	X^2^value	Pvalue
Oral ulcer	24 (92.31)	16(88.89)	0.150	0.698
Cancrum pudenda	15(57.69)	8(44.44)	0.748	0.541
Fever	11(42.31)	7(38.89)	0.052	1.000
Ophthalmitis	6(23.08)	7(38.89)	1.278	0.323
Intestinal BD	1(3.85)	1(5.56)	0.070	1.000
**DVT**	**10(38.46)**	**2(11.11)**	**4.359**	**0.046**
**Erythema nodosum**	**9(34.62)**	**1(5.56)**	**5.889**	**0.030**
AECA positivity	8(30.77)	3(16.67)	1.128	0.288
ANA positivity	4(15.38)	2(11.11)	1.281	0.527

DVT, deep vein thrombosis.

## Discussion

Immune complexes (ICs) [23] are products of reactions that involve noncovalent interactions between foreign antigens or autoantigens and antibodies. Circulating ICs (CICs) are possibly pathogenic unless they are removed by phagocytosis. Identification of antigens incorporated into CICs provides information that may be helpful in developing diagnostic and treatment strategies for autoimmune diseases. Recently, novel proteomic strategy (immune complexome analysis) enabled identification and profiling of antigen components in CICs[13] and make CIC proteomic analysis an effective way of exploiting new disease associated biomarkers in autoimmune diseases.

Behcet’s disease (BD) is a multisystem involved vasculitis with unclear etiology, and immune complexes have been reported to be deposited in affected tissues during disease development, which suggested the involvement of autoimmunity in this disease [24,25]. Since few autoantibodies have been proved to be specifically associated with BD, comprehensive profiling of antigens incorporated in CICs from BD patients may be an effective way of searching novel autoantigens or autoantibodies as diagnostic biomarkers. In this study, we applied high resolution mass spectrometry with nano-LC combined with Orbitrap Q Exactive mass spectrometer to screen CICs incorporated potential autoantigens of BD patients. A total of 17 potential autoantigens were identified in CICs of BD patients, including alpha-enolase, anti-streptococcal/anti-myosin immunoglobulin lambda light chain variable region, tubulin-α-1c, et al (Table 1), most of which were first reported in this study. It is noteworthy that these antigens were involved in physiological process including immune response, cell structure integrity, coagulation cascade, et al, which implicated that the corresponding autoantibodies against these antigens might interfere these physiological process above and lead to cell/tissue damages or pathogenic abnormalities in these process. Besides the identification of a series of potential BD associated autoantigens by immune complexome analysis, we further confirmed that tubulin-α-1c was a specific autoantigen of BD by indirect ELISA assay to test the anti-tubulin-α-1c autoantibodies, which were significantly increased in BD patients than those in disease and healthy controls and showed promising diagnostic performance. This result confirmed the feasibility and reliability of immune complexome analysis on novel biomarker screening in autoimmune diseases.

It is generally believed that generation of autoantibodies is critical for development of many autoimmune diseases. However, unlike many autoimmune diseases, BD cannot be definitively diagnosed using those commonly detected autoantibodies in autoimmune vasculitis, such as antinuclear antibody (ANA) or anti-neutrophil cytoplasmic antibodies (ANCA). Previous studies has proved that anti-endothelial cell autoantibody (AECA) contributed to the vascular damage of BD [26–28]. However, AECA positivity rates were relatively low in different BD patient cohorts, which were 13.1%, 26%, and 47.5% in Turkish, Spanish, and Chinese BD patients respectively, and AECA was also present in other diseases or in healthy controls, making sensitive and specific diagnosis of BD by AECA very challenging [28, 29]. Nowadays, the diagnosis of BD is mainly dependent on clinical criteria and the lack of reliable serological markers for accurate diagnosis of BD restrains the improvement of BD management. Therefore, in this study, identification of anti-tubulin-α-1c as a novel biomarker for BD diagnosis with improved performance may be promising for better management of BD.

Many investigations have been carried out on the relationship between tubulin family molecular and autoimmune conditions. α-tubulin might be a novel marker autoantigen for a neuropsychiatric manifestation at least in a subgroup of patients with SLE[18]. Goers et al. demonstrated that antibodies against the K-α-1 tubulin which is highly homologous to tubulin-α-1ccould be defined in human lung transplant recipients undergoing BOS. These antibodies bound to endothelial cells and the specific ligation results in increased expression of fibrogenic growth factors, activation of cell cycle signaling and fibro-proliferation [19, 20]. Tubulins mainly play their physiological roles in cellular structure maintenance, GTPase activity and intracellular movement as components of cytoskeleton [21]. Earlier studies have identified autoantibodies against K-α-1 tubulin and implicated that this protein could reach cell surface and was immunogenic under specific circumstances [22,30–31]. It was also hypothesized that tubulin-α-1c might stimulate the expression of VEGF and damage endothelial cells in vasculitis and thrombosis [32].

In the present study, we showed that anti-tubulin-α-1c autoantibodies were correlated with complications of deep venous thrombosis and erythema nodosum in BD. Meanwhile, levels of anti-tubulin-α-1c were positively correlated with BD disease activity. Although our result showed that anti-tubulin-α-1c did not totally overlap with AECA, it is still possible that anti-tubulin-α-1cautoantibodies might target the endothelial cells expressing tubulin-α-1c and eventually lead to endothelial cell damage and local vascular inflammation in BD. Alternatively, it is also possible that the anti-tubulin-α-1c could be secondary products of BD chronic inflammation. It is necessary to elucidate the pathological roles of tubulin-α-1c and its autoantibodies in BD in future investigations.

In conclusion, this study has successfully set up the immune complexome analysis method of BD patients by high resolution mass spectrometry, and identified 17 potential autoantigens in the serum of BD patients. Among these candidate autoantigens, tubulin-α-1c was proved to be an autoantigen of BD and its autoantibody was elevated in BD with promising diagnostic power. The anti-tubulin-α-1c autoantibody was positively correlated with disease activity and the incidence of DVT and Erythema nodosum in BD, which implicated anti-tubulin-α-1c might be involved in the vascular damage pathogenesis of BD. However, there were still obvious limitations in this study. First, because of the low incidence of BD in China [4], the number of BD patients recruited from our clinical center was limited in this study, and it might restrain the elucidation of clinical relevance of anti-tubulin-α-1c due to lack of statistical power. It will be necessary to recruit more BD patients from multiple clinical centers in our future studies on anti-tubulin-α-1c. Another weakness of this study is that we did not reveal the exact pathogenesis of anti-tubulin-α-1c in BD, especially how it affected vascular endothelial cells, which should also be revealed in the future investigations. Previous studies have shown that the deposition of immune complexes, which were formed by vascular autoantigens such as histones, ribosomes, fibronectin, etc. and their autoantibodies, were able to cause vascular inflammation, and lead to exacerbated local inflammation within vascular endothelium [33]. As tubulin-α-1c is a common cellular structural protein and could be exposed from damaged cells in autoimmune conditions, we proposed that similar to the pathological mechanism above, anti-tubulin-α-1c might form immune complex with its exposed antigen, leading to immune complex deposition and subsequent vascular inflammation and tissue destruction. Anti-tubulin-α-1c might also bind to its antigen on vascular cells and induce inflammatory response and exacerbate tissue damage. Previous study showed that binding of the anti-K-α-1 tubulin to epithelial cells resulted in the increased expression of TCF5 [19], which induced downstream proinflammatory cytokines such as VEGF and sustained local tissue inflammation. However, whether the exact role of the pathogenesis of anti-tubulin-α-1c in BD would follow the proposed mechanism above or not is still to be determined by future investigations, which will address the pathogenic roles of anti-tubulin-α-1c in BD.

## Supporting information

S1 Table The international criteria for BD in 1990. (PDF)

## Abbreviations

BD: Behcet’s disease
ELISA: Enzyme-Linked Immunosorbent Assays
MALDI-TOF: Matrix-Assisted Laser Desorption/Ionization Time of Flight
SSc: Systemic sclerosis
OA: Osteoarthritis
BD: Behcet’s disease
HC: Healthy control
CRP: C-reactive protein
ESR: Erythrocyte sedimentation rate
2-DE: Two dimensional electrophoresis
PBMC: peripheral blood mononuclear
