# Supplementary material for: Circulating immune complexome analysis identified anti-tubulin-α-1c as an inflammation associated autoantibody with promising diagnostic value for Behcet’s Disease
Source: PLoS One. 2018 Jun 14;13(6):e0199047. doi: 10.1371/journal.pone.0199047 (PMC6002243; doi:10.1371/journal.pone.0199047)
Supplement: S1 Table — (PDF) [file pone.0199047.s001.pdf]

S1 Table：The international criteria for BD in 1990.

1. recurrent oral ulcers	It was observed by the physician or told by the patient that he had ulcer, which had been repeated three times in a year.
2. recurrent genital ulcer	It was observed by the physician or the patient that the genitals had afuta ulcer or scar, especially the male.
3. opthalmopathy	Anterior and posterior uveitis, cell infiltration and retinal vasculitis could be seen in the glass when the eyes were examined by the slit lamp.
4. skin lesion	lesions of erythema nodules, pseudofolliculitis, purulent pimples, acne itchy rash（no glucocorticoids were taken）
5.the needle test was positive.	the oblique needle(aseptic 20 or smaller needles) was inserted into the skin, and the results were determined by the doctor after 24 to 48 hours.（a red papule or pustule and the size of a rice grain）

Judgment: patients with recurrent oral ulcer and any two of the remaining four items can be diagnosed as BD.
